# Greater Concentrations of IGF Binding Protein-2 after Bariatric Surgery Compared with Diet

**DOI:** 10.1210/jendso/bvaf218

**Published:** 2026-01-29

**Authors:** Chino Aneke-Nash, Sarah Borden, Emily G Werth, Jamie Leskowitz, Rajasekhar Ramakrishnan, Jorge Arriaza Sagredo, Tirissa J Reid, Abraham Krikhely, Marc Bessler, Lewis M Brown, Judith Korner

**Affiliations:** Digestive and Liver Disease, Department of Medicine, Columbia University Vagelos College of Physicians and Surgeons, New York, NY 10032, USA; Endocrinology, Department of Medicine, Columbia University Vagelos College of Physicians and Surgeons, New York, NY 10032, USA; Quantitative Proteomics and Metabolomics Center, Department of Biological Sciences, Columbia University, New York, NY 10027, USA; Endocrinology, Department of Medicine, Columbia University Vagelos College of Physicians and Surgeons, New York, NY 10032, USA; Department of Pediatrics, Columbia University Vagelos College of Physicians and Surgeons, New York, NY 10032, USA; Endocrinology, Department of Medicine, Columbia University Vagelos College of Physicians and Surgeons, New York, NY 10032, USA; Endocrinology, Department of Medicine, Columbia University Vagelos College of Physicians and Surgeons, New York, NY 10032, USA; Department of Surgery, Columbia University Vagelos College of Physicians and Surgeons, New York, NY 10032, USA; Department of Surgery, Columbia University Vagelos College of Physicians and Surgeons, New York, NY 10032, USA; Quantitative Proteomics and Metabolomics Center, Department of Biological Sciences, Columbia University, New York, NY 10027, USA; Endocrinology, Department of Medicine, Columbia University Vagelos College of Physicians and Surgeons, New York, NY 10032, USA

**Keywords:** bariatric surgery, low-calorie diet, insulin-like growth factor binding protein, adiponectin, leptin, obesity

## Abstract

**Context:**

Bariatric surgery causes greater sustained weight loss (WL) and metabolic improvements compared to lifestyle modification. It remains unclear which metabolic changes are solely attributable to WL and which also involve WL-independent changes.

**Objective:**

The objective of this study was to quantify changes in the adiponectin/leptin ratio and IGF binding protein 2 (IGFBP-2), both markers of metabolic disease.

**Methods:**

Adults with body mass index ≥35 kg/m^2^ underwent a 12-week 800 kcal/day low-calorie diet (LCD; n = 20), sleeve gastrectomy (n = 18), or Roux-en-Y gastric bypass (n = 10) and were studied at baseline [time 1(T1)], early weight loss [time 2 (T2)], and 1 year [time 3 (T3)]. As outcomes were similar between surgeries, the groups were combined for analysis.

**Results:**

The LCD and surgery groups had similar median WL of 15% at T2 (*P* = .72), achieved in 90 vs 48 days, respectively. The LCD group maintained WL at T3 whereas WL was 30% after surgery. At T2, the adiponectin/leptin ratio increased similarly; however, IGFBP-2 increased to a greater extent after surgery, 203 ng/mL (169-259) vs LCD, 153 (110-181; *P* = .028). Further WL after surgery at T3 resulted in a more marked increase in the adiponectin/leptin ratio, yet IGFBP-2 levels remained the same.

**Conclusion:**

IGFBP-2 levels and the adiponectin/leptin ratio improve after WL. The greater increase in IGFBP-2 levels after surgery compared with LCD may have long-term beneficial effects that appear to be partly independent of the degree of weight reduction.

Weight loss (WL) improves or prevents obesity-related comorbidities such as insulin resistance, type 2 diabetes mellitus, and cardiovascular disease [1-4]. Unfortunately, WL through lifestyle interventions has a high degree of weight recurrence [5, 6]. Metabolic and bariatric surgery leads to sustained WL and metabolic improvements in comparison to lifestyle modification with or without medical management [7-13]. Improvements in parameters such as insulin sensitivity after bariatric surgery are likely due to a combination of calorie restriction, WL, and WL-independent changes [14, 15]. We and others have shown similar changes in insulin sensitivity and β-cell function in patients with and without type 2 diabetes after Roux-en-Y gastric bypass surgery (RYGB) compared with equivalent WL after diet [16, 17]. However, bariatric surgery is associated with profound changes in circulating levels of hormones secreted from the gastrointestinal tract that make up the “gut-brain” and “enteroinsular” axes that are not observed after diet or laparoscopic adjustable gastric banding [18-26]. Changes in signaling molecules unique to surgery likely provide metabolic benefits in addition to calorie restriction alone [27].

To further elucidate the pathways that may explain the sustained therapeutic benefit of bariatric surgery vs caloric restriction, we utilized proteomics to identify proteins in human plasma that change in response to WL achieved through low-calorie diet (LCD) vs RYGB and vertical sleeve gastrectomy (SG). Preliminary unpublished results in 16 subjects (8 LCD, 4 RYGB, 4 SG) showed increases in IGF binding protein-2 (IGFBP-2) and adiponectin, but it was unclear how changes compared between groups, particularly at equivalent WL. IGF-1 stimulates the differentiation of preadipocytes and mediates the growth-promoting effects of GH [28]. IGF-1 circulates in the serum bound to 6 IGF binding proteins (IGFBPs). IGFBP-2 is the second most abundant IGF binding protein and has roles independent of its function as a binding protein and can induce the translocation of GLUT-4 receptors for glucose uptake in adipocytes, a role that is independent of its function as a binding protein [29]. Data from animal studies suggest a role in glucose homeostasis and insulin resistance as well as inhibitory effects toward adipogenesis and visceral lipogenesis [30-32]. IGFBP-2 has been shown in population studies to be a robust marker of incident diabetes in adults [33, 34]. Adiponectin increases insulin sensitivity, reduces hepatic gluconeogenesis, stimulates fatty acid catabolism, and increases energy expenditure through peripheral and central mechanisms [35]. Leptin regulates body weight through modulation of energy expenditure, food intake, and fatty acid oxidation [36] While individuals with obesity typically have low levels of adiponectin and high leptin levels, evidence suggests that the adiponectin/leptin ratio (A/LR)is a better marker of metabolic dysfunction since the ratio decreases with increasing adiposity and low levels may be a marker of adipose tissue dysfunction [37, 38].

The objective of this study was to follow up on our preliminary findings in a cohort using specific hormone assays to determine WL-dependent and independent changes in the aforementioned proteins and whether such changes differ between nonsurgical and surgically induced WL.

## Materials and Methods

### Study Participants

This study was approved by the Columbia University Institutional Review Board and was registered at www.ClinicalTrials.gov (NCT03371368). Oral and written informed consent was obtained from all participants. Three groups of adults ages 21 to 70 years were enrolled with body mass index (BMI) ≥ 35 kg/m^2^, who were either willing to follow a LCD (n = 20) or who were scheduled to undergo either SG (n = 18) or RYGB (n = 10). The choice of bariatric procedure was based on patient and surgeon preference and performed as described [18]. An additional group of healthy lean individuals (n = 12) with BMI from 18.5 to 24.9 kg/m^2^ was also enrolled. The main exclusion criteria were the presence of type 2 diabetes, major cardiovascular disease such as history of myocardial infarction or arrythmias, glomerular filtration rate < 60 mL/min/1.73 m^2^, liver transanimases >3 times upper limit of normal, current use of psychiatric or neuroleptic medication, the use of medications that might affect body weight, or weight change of > 5% body weight within 3 months prior to enrollment.

### Study Design

All participants were studied at baseline prior to WL intervention [time 1 (T1)] and after 12 weeks on the LCD or at approximately 15% total body WL after surgery [time 2 (T2)]. The objective was to have the 3 study groups mean-matched for percentage WL at T2. Participants were studied again at 1 year after the start of the intervention [time 3 (T3)]. Participants in the lean control group were studied once. Subjects in the LCD cohort had weekly visits with the registered dietitian for the first 12 weeks, every 2 weeks for another 12 weeks, and monthly visits for the remainder of the study until 1 year. For the first 12 weeks, participants consumed 5 to 6 meal replacements/day, totaling 800 to 960 calories per day (Optifast, Novartis, Minneapolis, MN, USA; 160 kcal per replacement, 50% carbohydrate, 35% protein, 15% fat). After 12 weeks, participants were given instructions on a partial meal replacement transition diet: 3 meal replacement products, 1 meal (455-560 kcal), 1 snack (160 kcal) for 1 month; 2 meal replacement products, 2 meals, 1 snack for 2 months. For the remaining 6 months, subjects were instructed to consume 1200 to 1500 calories/day. All meal replacements were provided. Out of clinical necessity, the dietary counseling differed between the LCD and surgery groups as the latter needed to escalate the diet postoperatively from liquid to solid over a 6-week period. It would not have been feasible to mimic this diet in the LCD group. Surgery patients were contacted regularly to report their body weight with the objective of having a second clinical visit at approximately 15% WL. At each clinical visit, weight and waist circumference were measured and fasting venous blood samples were collected. After centrifugation at 4 °C, serum and plasma were stored at −80 °C. All assay measures were done using these fasting samples.

### Assays

Serum IGFBP-2 levels were quantified by ELISA (R&D Systems Cat# DGB200, RRID:AB_3717941, https://www.antibodyregistry.org/AB_3717941). Assay sensitivity was 0.04 ng/mL, and intra- and interassay coefficient of variation (%CV) were 3.6% to 5.0% and 4.5% to 7.6%, respectively. Serum IGFBP-3 levels were quantified by ELISA (R&D Systems Cat# DGB300, RRID:AB_2915952, https://www.antibodyregistry.org/AB_2915952). Assay sensitivity was 0.05 ng/mL, and intra- and interassay %CV were 2.3% to 4.8% and 5.4% to 8.0%, respectively. Serum IGF-1 levels were quantified by ELISA (R&D Systems Cat# DG100B, RRID:AB_2915951, https://www.antibodyregistry.org/AB_2915951). Assay sensitivity was 0.01 ng/mL, and intra- and interassay %CV were 4.0% to 4.5% and 5.7% to 6.2%, respectively. Serum samples were treated as per the manufacturer's instructions to quantify levels of high molecular weight adiponectin by ELISA (Alpco Diagnostics Cat# 80-ADPHU-E01, RRID:AB_2892778, https://www.antibodyregistry.org/AB_2892778). Assay sensitivity was 0.034 ng/mL, and intra- and interassay %CV were 1.1% to 5.7% and 3.6% to 6.4%, respectively. Plasma leptin levels were quantified by ELISA (R&D Systems Cat# DLP00, RRID:AB_2783014, https://www.antibodyregistry.org/AB_2783014). Assay sensitivity was 7.8 pg/mL, and intra- and interassay %CV were 1.3% to 3.2% and 3.5% to 5.4%, respectively. Serum GH levels were quantified by Immulite Analyzer (Siemens Cat# LKGRH1, RRID:AB_3717947, https://www.antibodyregistry.org/AB_3717947). Assay sensitivity was 0.01 ng/mL, and intra- and interassay %CV were 5.3% to 6.5% and 5.5% to 6.2%, respectively. Serum insulin levels were quantified by Immulite (Siemens Cat# KLIN1, RRID:AB 2750939, https://www.antibodyregistry.org/AB_2750939). Assay sensitivity was 2 μIU/mL, and intra- and interassay %CV were 5.2% to 6.4% and 5.9% to 8.0%, respectively. Plasma glucose was measured by the hexokinase method.

### Statistical Analysis

**Figure 1.**
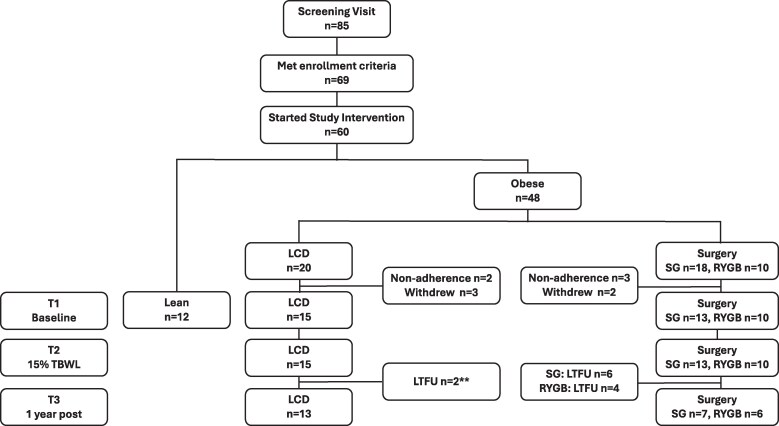
CONSORT flow diagram. Only participants who completed T1 and T2 were included in the data analysis. Abbreviations: LTFU, lost to follow-up; T1, time 1; T2, time 2.

The CONSORT flow diagram for the study is presented in Fig. 1. Only participants who had data for both T1 and T2 visits were analyzed: of the 20 participants who started the LCD, 15 were available for study (3 withdrew and 2 were not analyzed due to protocol nonadherence). Of the 18 participants who underwent SG, 13 were studied (2 withdrew and 3 were lost to follow-up); all 10 participants who underwent RYGB were analyzed at T2. The number of participants available for analysis at T3 were as follows: 13 LCD, 7 SG, and 6 RYGB. The power analysis was based on the primary endpoint of the study, which was to compare changes in the plasma proteome between the different intervention groups. For the primary endpoint, differences in glucagon-like peptide 1 values obtained from our prior studies were used to determine sample size, which was calculated to be 12 subjects in each group to achieve over 95% power to detect expected differences in glucagon-like peptide 1 values. The Shapiro-Wilk test was used to assess for normality of all variables [39]. Baseline levels among obese and lean groups were analyzed using Student's *t*-test for parametric data and the Dunn's all-pairs test, which generalizes Wilcoxon rank-sum test for nonparametric data points. The Bartlett test for homogeneity of variances was used to assess the assumption of equal variances, and when not met, the Welch's *t*-test was used instead. Similar analyses were carried out for baseline comparison of individuals in the LCD vs surgery groups. Absolute changes in hormone levels over time were analyzed using repeated measures ANOVA, and *P*-values for paired and unpaired comparisons of the absolute changes are reported. For the repeated measures analyses, variables were either log-transformed or square-root transformed if the normality assumptions were not met. Hormone levels at T2 among the LCD and surgery groups were compared to measurements from lean individuals using the methods stated previously. Correlations between WL percent from T1 to T2 and from T1 to T3 with change in hormone levels among the LCD vs surgery groups were analyzed using Pearson correlation or Spearman correlation when normality assumption was not met. Insulin resistance was calculated using the homeostatic model of assessment (HOMA-IR) [40]. Sensitivity analysis including only those with complete data across all 3 visits. Statistical analyses were carried out using R (Version 4.3.1) with the cufunctions package [41]. All tests were 2-tailed, and a *P*-value of ≤.5 was considered statistically significant. Graphs were produced with GraphPad Prism.

## Results

Characteristics of 12 lean controls and 38 individuals with obesity are presented in Table 1. The 2 groups were similar in age and sex. There was a greater percentage of Hispanics in the group with obesity and a greater percentage of Asians in the lean group. Fasting glucose was similar in both groups, whereas hemoglobin A1c, insulin, HOMA-IR, and leptin were greater in the cohort with obesity. GH, IGF-1, and IGFBP-2 levels, as well as adiponectin and A/LR (measured in ng/mL for both proteins) were all significantly lower in the cohort with obesity. IGFBP-3 levels were not significantly different between the groups (*P* = .08).

**Table 1.** Baseline characteristics of lean controls and participants with obesity

Variable	Lean	Obese	*P*
Sex, n (F/M)	12 (7/5)	38 (29/9)	.24
Age (y)	37.2 (11)	41.3 (11.9)	.3
Ethnicity n (NH/H)	12 (11/1)	38 (20/18)	.002
Race n (W/B/A/O)	12 (6/1/5/0)	38 (21/15/0/2)	
Weight (kg)	69 (60-73)	121 (114-140)	<.0001
BMI (kg/m^2^)	23.0 (21.6-23.9)	43.2 (41.2-48)	<.0001
Waist (cm)	78.2 (5.94)	125.0 (13.4)	<.0001
Glucose (mg/dL)	91.8 (9.2)	96.4 (12.7)	.25
Insulin (μIU/mL)	3.6 (2.6-4.8)	16.4 (11-23.9)	<.0001
HOMA-IR	0.83 (0.55-1.21)	3.92 (2.72-5.83)	<.0001
HbA1c (%)	5.1 (5.1-5.2)	5.6 (5.4-5.9)	<.0001
GH (ng/mL)	1.6 (0.2-3.4)	0.2 (0.09-0.30)	.002
IGF-1 (ng/mL)	135.0 (39.7)	72.6 (29.3)	<.0001
IGFBP-2 (ng/mL)	235 (112-314)	83 (69-110)	<.0001
IGFBP-3 (ng/mL)	2210 (572)	1860 (586)	.08
Adiponectin (ng/mL)	2440 (2170-3130)	1320 (810-1920)	.002
Leptin (ng/mL)	5.4 (3.2-14.7)	66.2 (51.4-92.3)	<.0001
Adiponectin/leptin ratio	581 (288-690)	22 (13-31)	<.0001

Data are presented as mean (SD) or median (interquartile range) when not normally distributed.

Abbreviations: A, Asia; B, Black; BMI, body mass index; F, female; H, Hispanic; HbA1c, hemoglobin A1c; HOMA-IR, homeostatic model assessment of insulin resistance; IGFBP, IGF binding protein; M, male; NH, non-Hispanic; O, other; W, White.

*P* represents comparison of groups with unpaired *t*-test.

A comparison of LCD and surgery group over time is presented in Table 2. All parameters studied were similar both before and after WL in the SG and RYGB groups with the exception of baseline GH levels (0.23 vs 0.61 ng/mL; *P* = .04); therefore, the surgery groups were combined for analysis. At baseline, there were no significant differences in age or sex, although a lower percentage of individuals in the LCD group were of Hispanic origin (20% vs 65%; *P* = .005). As per study design, both groups had similar mean WL of approximately 15% at the first interval follow-up at T2 (*P* = .72); however, the LCD group achieved the WL in a longer time period than the surgery group (90 days, interquartile range: 85-95 vs 48 days, interquartile range: 45-91, *P*  *=* .0001). The LCD group maintained 15% WL at 1 year, although there was some variability in the trajectory of WL, whereas there was continued WL in all surgery participants that reached a greater group mean of 30% WL (*P* < .001; Table 2).

**Table 2.** Characteristics before and after weight loss with LCD or surgery

	LCD	Surgery	*P*
Sex, n (F/M)			
T1 and T2	15 (12/3)	23 (17/6)	.68
T3	12 (10/2)	13 (10/3)	.69
Age (years)	44.5 (10.8)	39.2 (12.4)	.19
Ethnicity, n (NH/H)			
T1 and T2	15 (12/3)	23 (8/15)	.005
T3	12 (9/3)	13 (3/10)	.69
Race, n (W/B/O)			
T1 and T2	15 (6/9/0/0)	23 (15/6/0/2)	
T3	12 (5/7)	13 (8/5)	
Weight loss (%)			
T2-T1	14.9 (2.7)	14.5 (4.04)	.72
T3-T1	14.6 (6.8)	30.2 (5.78)	<.0001
BMI (kg/m^2^)			
T1	45.1 (4.3)	44.4 (5.5)	.45
T2	38.3 (3.6)***	37.9 (4.9)***	.82
T3	38.6 (5.9)***	30.9 (4.4)***^c^	<.0001
Waist (cm)			
T1	127 (16.7)	124 (10.9)	.54
T2	112 (12.1)***	113 (12.6)***	.74
T3	110 (12.5)***	92 (9.0)***^c^	<.001
Glucose (mg/dL)			
T1	97.4 (15.1)	95.8 (11.2)	.72
T2	88.3 (9.9)**	87.8 (12.1)**	.89
T3	92.4 (5.0)	83.8 (5.3)***	.09
Insulin (μIU/mL)			
T1	17.6 (10.8 - 23.6)	14.9(11.1 - 23.9)	.77
T2	6.9 (4.4 - 11.2)***	8.1(6.1 - 10.6)***	.61
T3	7.7 (4.4 - 13.3)***	4.9 (2.8 - 7)***^a^	.07
HOMA-IR			
T1	3.8 (2.9 - 5.8)	4.0 (2.7 - 5.5)	.75
T2	1.5 (1.0 - 2.5)***	1.7 (1.2 - 2.5)***	.66
T3	1.7 (1.0 - 3)***	1.0 (0.6 - 1.5)***^b^	.05
GH (ng/mL)			
T1	0.1 (0.09 - 0.2)	0.2 (0.1 - 0.4)	.15
T2	0.5 (0.2 - 0.9)***	0.6 (0.2 - 1.2)***	.71
T3	0.6 (0.3 - 0.9)***	1.2 (0.4 - 1.8)***	.06
IGF-1 (ng/mL)			
T1	76 (50 - 111)	66 (47 - 85)	.13
T2	87 (69 - 123)*	50 (37 - 74)	<.001
T3	117(66 - 145)**	85(64 - 93)**^c^	.15
IGFBP-2 (ng/mL)			
T1	92 (75 - 113)	77 (68 - 107)	.65
T2	153(110 - 181)**	203 (169 - 259)***	.028
T3	125 (89.9 - 142)	189 (119 - 234)***	.005
IGFBP-3 (ng/mL)			
T1	1680 (556)	1980 (585)	.12
T2	1720 (557)	1860 (598)	.49
T3	1670 (424)	1900 (567)	.08
Adiponectin (ng/mL)			
T1	1510 (888 - 2130)	1170 (690 - 1680)	.22
T2	1640 (872 - 2160)	1420 (1050 - 2040)***	.89
T3	2080 (1320 - 3200)***^c^	2270 (1890 - 2810)***^c^	.42
Leptin (ng/mL)			
T1	69.6 (20.7)	70.3 (37.2)	.94
T2	36.0 (17.9)***	27.6 (16.6)***	.29
T3	46.0 (21.5)**	16.6 (10.2)***	.003
Adiponectin/leptin ratio			
T1	26 (16 - 32)	19 (14 - 28)	.64
T2	64 (42 - 71)***	61 (38 - 125)***	.14
T3	54 (37 - 73)***	174 (101 - 321)*** *^c^*	<.0001

Table 2. Levels of biomarkers during the study period among those who underwent a low calorie diet vs those who underwent surgery

Abbreviations: A, Asian; B, Black; BMI, body mass index; F, female; H, Hispanic; HOMA-IR, homeostatic model assessment of insulin resistance; IGFBP, IGF binding protein; LCD, low-calorie diet; M, male; NH, non-Hispanic; O, other; T1, time 1; T2, time 2; T3, time 3; W, White.

Data are presented as mean (SD) or median (interquartile range) when not normally distributed.

*P* represents comparison of LCD and surgery group with unpaired *t*-test.

*P < .05; **P < .01; ***P < .001 for within group paired *t*-test between T2 and T1 and between T3 and T1.

^*a*^ *P* < .05; ^b^*P* < .01; ^c^*P* < 0.001 for within group paired *t*-test between T3 and T2.

**Figure 2.**
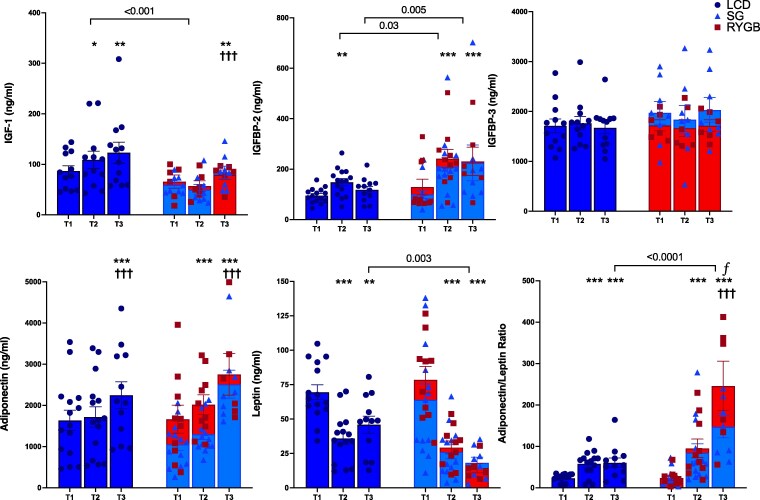
Biomarker levels over time. *P* > .05. ^ƒ^Adiponectin/leptin ratio contains an outlier value >900 that is not depicted in the figure. *P < .05, **P < .01, ***P < .001 for within group paired *t*-test between T2 and T1 and between T3 and T1. ^†^*P* < .05, ^††^*P* < .01, ^†††^*P* < .001 for within-group paired *t*-test between T3 and T2. Abbreviations: ns, nonsignificant; T1, time 1; T2, time 2; T3, time 3.

At similar WL, there were significant decreases in fasting glucose, insulin, and HOMA-IR that did not differ between groups. GH increased to a similar extent in both groups at T2 and progressively increased with further WL at 1-year postsurgery. While IGF-1 progressively increased with time after LCD, there was no significant change initially after surgery (*P* = .06) and a blunted increase in comparison to LCD group at 1 year (group × time *P* < .001 and *P* = .15 at T2 and T3, respectively; Fig. 2). IGFBP-2 levels increased significantly at T2 in both groups; however, the surgery group had a 2.5-fold greater increase (*P* = .074), resulting in significantly greater levels compared with the LCD group (*P* = .028). With further WL after surgery at T3, IGFBP-2 did not continue to increase. Changes in IGFBP-2 did not correlate with percentage WL at any time point in either group (*P* > .05 for all time points; correlations at T2 shown in Fig. 2). Changes in IGFBP-2 did not correlate with changes in insulin, glucose, waist circumference, or HOMA-IR (data not shown). Greater increases in IGFBP-2 levels at T2 were significantly correlated with decreases in waist-hip ratio in the LCD group and approached significance in the surgery group (LCD: r = −0.64, *P*  *=* .03, Surgery: r = −0.59, *P*  *=* .07). The changes did not vary by sex (*P*  *>* .05) but were more pronounced for those above the median age of 39 (*P*  *<* .0001). These results did not change after analysis was restricted to only those with complete data across all 3 visits. There were no significant changes in IGFBP-3 within each group at any time point.

**Figure 3.**
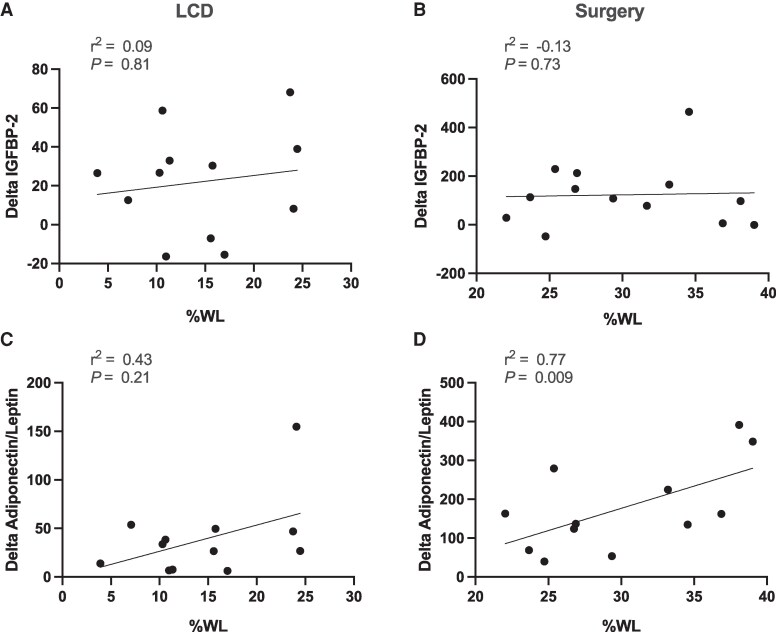
Correlations between changes in IGFBP-2 and adiponectin/leptin ratio with percentage WL at T2. Abbreviations: IGFBP-2, IGF binding protein 2; T2, time 2; WL, weight loss.

At T2, adiponectin levels increased significantly only after surgery. Both groups exhibited the greatest increases at T3. Although the change in levels was greater after surgery (*P* = .009) with twice as much percentage WL, absolute levels were not different compared with the LCD group (*P* = .42). Leptin levels decreased similarly from T1 to T2 between groups (*P* = .28) and continued to decrease with further WL at T3 after surgery when compared with LCD (*P* = .003). Of note, the A/LR increased similarly in both groups at T2. The ratio remained the same from T2 to T3 in the LCD group but continued to increase with further WL after surgery, resulting in an approximately 3-fold greater level compared with LCD (*P* < .0001). These results were similar when analysis was restricted to only those with complete data across all 3 visits, especially for the surgery group. The change in the ratio from T1 to T3 after surgery correlated with percentage WL (r^2^ = 0.77; *P* = .009), but this relationship was not statistically significant in the LCD group (r^2^ = 0.43; *P* = .21) (Fig. 3). Changes in A/LR did negatively correlate with HOMA-IR at T2 (r = −0.67, *P*  *=* .03). Changes in A/LR did not vary by sex or median age.

At the end of the study period, BMI in the surgery group was still greater compared with lean controls (*P* < .0001) yet HOMA-IR (*P* = .69), IGFBP-2 (*P* = .71), and A/LR (*P* = .12) were similar. In contrast, HOMA-IR was greater (*P* = .04), and IGFBP-2 (*P* = .02) and A/LR (*P* < .0001) were significantly lower in the LCD group compared with lean controls.

## Discussion

Metabolic and bariatric surgery improves metabolic risks through weight-dependent and weight-independent mechanisms, although the clinical contribution of the latter remains controversial [14]. Pinpointing the factors involved in each mechanism has proven challenging due to the complex interplay of pathways that change with both WL and surgical manipulation of the gastrointestinal tract. The main objective of this study was to confirm preliminary unpublished results of changes associated with WL using proteomic analysis and to compare such changes between LCD and bariatric surgery using ELISAs specific for each protein. Participants were evaluated after achieving a group mean WL of 15% body weight and again 1 year later to distinguish hormonal changes attributable to surgery vs WL effects alone. Weight change in the LCD group varied between individuals throughout the 1-year follow-up, although the mean change for the group remained the same, whereas the surgery group doubled their WL to 30% at T3. Significant changes in the IGF axis occurred after WL; levels of GH, IGF-1, and IGFBP-2 increased after WL, with surgery resulting in greater levels in IGFBP-2 compared to LCD. No changes were noted in IGFBP-3 levels in either group. Leptin levels decreased significantly in both groups, while adiponectin levels as well as the A/LR increased, with more pronounced changes seen in the surgery group after greater WL.

IGFBP-2 plays a critical role in metabolic disorders such as obesity and insulin sensitivity through both IGF-dependent and independent pathways [34]. Low levels of IGFBP-2 are associated with obesity, insulin resistance, gestational and type 2 diabetes, deleterious lipid profile, and metabolic syndrome [34]. Consistent with metabolic dysregulation, such as higher insulin resistance as measured with HOMA-IR, IGFBP-2 levels in our cohort with obesity at baseline were lower compared with the lean controls. While IGFBP-2 levels at baseline were similar between the LCD and surgery groups, treatment with surgery resulted in greater IGFBP-2 levels than diet when both groups achieved a similar median 15% WL. Although the LCD group lost the weight in a longer time period than the surgery group, at 1-year follow-up, there was no further increase in IGFBP-2 levels despite continued WL after surgery, suggesting that the changes are related to the WL method, rather than the timing of the WL. The finding that both groups exhibited increases suggests a WL-dependent change; yet the observation that surgery induced a greater increase in IGFBP-2 levels at T2 suggests that increased levels are in part independent of WL. Furthermore, the finding that these levels did not increase further upon doubling the percentage WL at T3 indicates that there may be a plateau effect that is maximal early after surgery. Additionally, these changes correlated with decreases in waist-hip ratio at T2 that was significant for the LCD group mostly, suggesting that changes in IGFBP-2 after surgery may have additional mechanisms independent of the additional WL noted in the surgery group at T2. While age was not significantly different at baseline, the observed changes in IGFBP-2 were greater in those above the median age of 39; however, the relatively small sample size limits meaningful clinical conclusions about this finding.

Increased IGFBP-2 levels after WL align with previous research showing increased levels after surgery [42-46], diet-induced WL [47], and a lifestyle modification program [48]. As shown in our study, similar increases in IGFBP-2 levels were observed after SG and RYGB at 3 months postsurgery, without any further increases at 1 year [44]. Notably, these studies did not concurrently evaluate subjects achieving similar WL with different modalities. One study identified IGFBP-2 as a target protein that increased comparably during both caloric restriction and bariatric surgery; however, this comparison relied on Mendelian randomization estimates rather than direct measurements of IGFBP-2 levels [49]. Changes in IGFBP-2 levels after bariatric surgery are durable and remain elevated 12 years postprocedure [50]. IGFBP-2 levels have been shown to be protective for diabetes risk in humans [33]. In adults, low IGFBP-2 and low adiponectin levels were strong independent predictors of glucose intolerance [51] and metabolic syndrome [52]. IGFBP-2 knockout mice display markedly less WL and greater food intake after RYGB, demonstrating an anorectic effect of this protein on feeding behavior [44]. It may be speculated that long-term durability of increased IGFBP-2 may contribute to greater WL and glucose control after surgery compared with lifestyle and medical management alone [7, 8] or decreased incidence of type 2 diabetes [53].

Our study did not reveal a mechanism that might explain the exaggerated increase in IGFBP-2, which occurred similarly after SG and RYGB compared with diet, although several hypotheses have been proposed by others and are consistent with our results. IGFBP-2 is regulated by multiple factors, including downregulation by GH receptors and upregulation by leptin [54, 55]. In the study by Dreyfuss et al, significant increases in IGFBP-2 up to 3 years after RYGB were demonstrated when compared with subjects enrolled in a diabetes and weight management clinic [46]. Their study revealed marked decreases in plasma GH receptor, a likely marker of decreased GH signaling, which in turn results in increased levels of IGFBP-2 and supports the hypothesis that decreased GH action (ie, GH resistance) contributes to postsurgical metabolic control in patients with diabetes. Ceccarini et al have suggested that increased leptin sensitivity may mediate increases in IGFBP-2 levels and exert an anorectic effect after RYGB [43]. It has also been proposed that bypassing a portion of the duodenum and proximal jejunum increases IGFBP-2 mRNA expression in the liver, as these changes were observed after surgery in mice but not in WL-matched sham-operated controls [44]. However, this mechanism alone would not explain similar increases after SG and RYGB in which part of the intestine is bypassed only in the latter procedure.

Previous research has documented an increase in IGF-1 levels following diet-induced WL [47, 56], behavioral health management [57], and bariatric surgery [58-60]; however, the changes after surgery are inconclusive [42, 61]. In our study, both groups displayed increases in IGF-1, although this only occurred in the surgery group at 1 year. Delayed increase in IGF-1 after surgery has been noted in 3 prior studies [58, 59, 61]. Disparate results between studies may be due in part to differences in study populations such as inclusion of only women [47, 56] or men [42], the presence of type 2 diabetes [58, 59], and differences in age or assay techniques. The increased levels of IGF-1 observed in our study may reflect a lowering of cardiometabolic disease risk in both groups [62-64].

The increase in A/LR in our study is in agreement with other reports after RYGB in patients with type 2 diabetes [65] and after diet and exercise intervention [66]. Here we show that this increase is similar after equivalent WL with LCD or surgery, and a further increase is driven by continued WL after surgery. It should also be noted that the assay used in this study measured the high molecular weight isoform of adiponectin, which is better correlated to insulin sensitivity than any of the other low molecular weight forms [38, 67]. Low A/LR is associated with inflammatory markers [68] and metabolic risk factors such as high triglycerides and insulin resistance [69]; thus, WL by any means would be expected to improve metabolic risk, yet greater long-term WL achieved with surgery likely results in additional benefits.

The main strengths of this study are that WL was achieved via both diet and 2 different types of surgery, and participants were evaluated at a time point with matched WL as well as after 1 year of follow-up. An advantage in the assessments was that patients with diabetes were excluded in order to eliminate the use of medications that might affect body weight, insulin sensitivity, or glucose metabolism. Limitations of the study include the lack of randomization to treatment, which is inherently prone to confounders that are unaccounted for and differences in the number of Asian and Hispanic participants in the lean controls and the group with obesity. Additionally, the diets differed among the LCD and surgery groups. While it is possible that differences in diet contributed to our findings, it seems unlikely as the surgery group maintained the increase in IGFBP-2 as their diet was liberalized by 1 year.

## Conclusion

WL via calorie restriction alone or in the setting of bariatric surgery has profoundly positive cardiometabolic effects. Increases in both the A/LR and IGFBP-2 levels were demonstrated in this study, where the former is primarily driven by WL and the latter is a result of both WL-dependent and independent mechanisms. Greater increases in IGFBP-2 levels may contribute to some of the lasting metabolic improvements, including protection from future metabolic disease, after bariatric surgery. Longer-term follow-up would be of interest to determine whether increased IGFBP-2 levels are maintained in individuals who experience weight regain after surgery.

## Data Availability

Some or all datasets generated during and/or analyzed during the current study are not publicly available but are available from the corresponding author on reasonable request.

## Clinical Trial Information

NCT03371368.
